# Non-Human Primate Models of HIV Brain Infection and Cognitive Disorders

**DOI:** 10.3390/v14091997

**Published:** 2022-09-09

**Authors:** Sarah J. Byrnes, Thomas A. Angelovich, Kathleen Busman-Sahay, Catherine R. Cochrane, Michael Roche, Jacob D. Estes, Melissa J. Churchill

**Affiliations:** 1 School of Health and Biomedical Sciences, RMIT University, Bundoora, VIC 3083, Australia; 2 The Peter Doherty Institute for Infection and Immunity, The University of Melbourne, Melbourne, VIC 3000, Australia; 3 Life Sciences, Burnet Institute, Melbourne, VIC 3004, Australia; 4 Vaccine and Gene Therapy Institute, Oregon Health & Science University, Portland, OR 97006, USA; 5 Oregon National Primate Research Centre, Oregon Health & Science University, Portland, OR 97006, USA; 6 Departments of Microbiology and Medicine, Monash University, Clayton, VIC 3800, Australia

**Keywords:** non-human primates, HIV, SIV, cognitive disorders

## Abstract

Human Immunodeficiency virus (HIV)-associated neurocognitive disorders are a major burden for people living with HIV whose viremia is stably suppressed with antiretroviral therapy. The pathogenesis of disease is likely multifaceted, with contributions from viral reservoirs including the brain, chronic and systemic inflammation, and traditional risk factors including drug use. Elucidating the effects of each element on disease pathogenesis is near impossible in human clinical or ex vivo studies, facilitating the need for robust and accurate non-human primate models. In this review, we describe the major non-human primate models of neuroHIV infection, their use to study the acute, chronic, and virally suppressed infection of the brain, and novel therapies targeting brain reservoirs and inflammation.

## 1. Introduction

Although viral suppression with antiretroviral therapies (ARTs) has significantly improved HIV prognosis, people with HIV (PWH) still require long-term treatment and have a higher risk of comorbid disease compared to people without HIV [1]. Specifically, 30–69% of ART-treated, virally suppressed PWH develop HIV-associated neurocognitive disorders (HANDs) [2,3,4,5]. HANDs cause cognitive and motor issues leading to reduced independence and challenges associated with job-related tasks, organizational skills, homemaking, medication adherence, and driving [6,7,8,9,10]. Whilst sustained ART suppression of viremia has dramatically reduced the frequency of severe HIV-associated dementia (HAD) and HIV encephalitis (HIVE), which affects 10–15% of unsuppressed individuals [11,12], the incidence of milder forms of HANDs (namely; asymptomatic neurocognitive impairment (ANI) and mild neurocognitive disorder (MND)) has increased in the post-ART era [13,14]. PWH also exhibit cognitive disease progression, continued loss in brain volume, and ongoing pathology that is compounded by age and not resolved by ART [15,16,17,18]. Therefore, as PWH age, it is estimated that HANDs will place an increasing burden on health resources, with an estimated annual cost of AUD 53 million by 2030 in Australia alone [19].

The mechanisms driving HANDs are unclear, partly due to a likely multifaceted disease pathogenesis which is difficult to define using ex vivo human studies alone. Therefore, the majority of our understanding of central nervous system (CNS) infection has been derived from autopsy material and cerebrospinal fluid (CSF) biomarker studies. It is thought that localized infection in the brain, shown to occur during acute infection, coupled with chronic systemic inflammatory mechanisms, may contribute to neuronal degradation and HAND pathogenesis. However, the paucity of brain tissue and the inherent inability to access longitudinal tissue samples in humans highlights the need for robust animal models to better define HAND pathogenesis.

Non-human primate (NHP) models offer unique insight into the underlying and governing mechanisms of neurocognitive disorders for many diseases in a manner not possible in humans. For instance, the spontaneous NHP model of multiple sclerosis (MS), a characteristically difficult disease to study at a cellular level in humans, mimics the immunopathology of humans and has corroborated the role of a virus in demyelinating diseases [20]. Recent advances and access to non-invasive approaches in such models also allow for the assessment of both the cellular and behavioral effects of disease including functional magnetic resonance imaging (MRI) and fluorodeoxyglucose-positron emission tomography (FDG-PET), thus providing translational evidence for humans. Therefore, animal models have allowed for significant advances in our understanding of these key steps in disease pathogenesis.

NHP models infected with simian immunodeficiency virus (SIV) are the most physiologically relevant model of HIV. NHPs infected with SIV show similar disease pathogenesis to HIV, including the development of acquired immune deficiency syndrome (AIDS), gastrointestinal damage and traditional AIDS-defining illnesses and comorbid disease ([21], as reviewed by [22]). Importantly, SIV infection can be controlled and suppressed with common ART regimens currently used in humans, therefore allowing for the long-term assessment of the effects of chronic ART-suppressed SIV infection, which reflects the majority of PWH worldwide. This allows for the assessment of SIV reservoirs in the body and analytical treatment interruptions required to assess HIV cure strategies. Importantly, SIV also enters the CNS early during infection and adaptations to promote neurotropism have resulted in advanced models of neuroHIV.

In this review, we will discuss the different NHP models of neuroHIV and their uses for studying viral entry and pathogenesis in the brain during acute, chronic, and virally suppressed SIV infection. We will further discuss the appropriateness of these models to study cognitive disorders associated with HIV and address the key unanswered questions in the field; namely,

- How and when does HIV enter the CNS?
- How and where does HIV establish and maintain viral reservoirs?
- Are reservoirs of HIV in the brain replication competent?
- What is the role of chronic systemic peripheral inflammation in CNS dysfunction?

These questions are exceptionally difficult to answer in vivo in humans alone, as unlike blood or peripheral tissue biopsies, sampling cannot be readily performed on the brain. Furthermore, intervention studies are difficult and, in some cases, unethical to perform. Therefore, physiologically relevant animal models are essential to answer these questions.

## 2. Non-Human Primate Models of NeuroHIV

NHP animal models are a powerful and versatile tool in the study of HIV infection and persistence in the brain. Rhesus, cynomolgus, or pigtailed macaques have physiology, immune system biology, neuroanatomy, and gastrointestinal tract (GIT) development and anatomy analogous to humans, and when infected with strains of SIV display remarkably similar infection and disease progression to HIV infection [23,24]. Importantly, SIV infection of NHPs recapitulates the key features of HIV transmission (including mucosal transmission), viral dissemination, receptor usage and cellular tropism, disease progression, pathology, and ART response (as reviewed in [22,25]). Long-term NHP studies also allow for the longitudinal assessment of SIV viremia, immune activation, and ongoing pathogenesis in tissue compartments such as the lymph nodes, GIT, CSF, and brain, which are not readily available from humans in most clinical studies. Importantly, different viral models have been developed to answer key questions in early infection events, chronic disease pathogenesis, and reservoir establishment/targeting in the brain. These are described in detail below (Table 1).

### 2.1. Accelerated CNS Infection

Accelerated CNS viral strains rapidly infect the CNS and typically result in SIV encephalitis (SIVE) and full immunosuppressive disease over a period of months. For instance, the infection of pigtailed macaques with both SIV/17E-Fr (neurotropic) and SIV/ΔB670 (immunosuppressive swarm) results in neurological disease in approximately 90% of animals within 85 days post inoculation [26,27,28]. The rapid rate of infection and virulence of this strain has been useful in that it allows for the characterization of early infection events and pathology in a significantly reduced time to CNS disease pathogenesis and a higher frequency of neurological disease compared to non-accelerated strains [28]. While this accelerated model of CNS infection is ideal for SIVE studies, utilizing ART reduces the incidence of SIVE, which necessitates a model of ART treatment in the HIV infection of the brain [29,30]. Notably, other strains of SIV described below can exhibit accelerated disease pathogenesis when host animals are CD8+ or CD4+ T-cell-depleted prior to infection, thereby allowing viral infection to overwhelm host immune responses [31]. Different NHP species also have increased susceptibility to SIV/17E-Fr + SIV/ΔB670, with pigtailed macaques developing SIVE at a higher frequency and a quicker rate than rhesus macaques (RMs) [32], highlighting the importance of selecting the most suitable animal model for the research question.

### 2.2. Neurotropic SIVs

Primate lentiviruses (i.e., HIV-1, HIV-2 and SIVs) are dual receptor viruses that use both the primary CD4 receptor and a coreceptor (primarily CCR5, CXCR4 or both) and by virtue of their ability to infect myeloid lineage cells as well as CD4+ T cells, establish infection within the CNS within 2 weeks post infection. NHPs infected with SIVmac251 or the molecular clone SIVmac239 will typically progress to AIDS within 1–2 years without ART, compared to 8–10 years for humans [33]. Animals infected with SIVmac251/239 develop neurological disease at a similar frequency to PWH, with approximately 25% of animals developing SIVE within 1–3 years post infection [34,35]. As such, these models are essential for the research of acute and chronic HIV infection and disease, the effect of chronic systemic inflammation, and viral reservoir persistence (latent and active) during long-term infection under suppressive ART. The depletion of CD8+ lymphocytes prior to SIVmac251/239 infection accelerates disease progression and severity, with up to 85% of animals developing SIVE within 6 months of infection [36]. While these models are beneficial to accelerate disease progression, they may not be suitable when assessing neuro-infection due to the critical role that CD8+ T cells play in targeting and killing virally infected cells in the blood and the CNS [37,38].

Similar to the CD8+ T cell depletion model, the depletion of CD4+ T cells prior to SIVmac251 inoculation accelerates disease progression and severity in the CNS due to productive microglia infection [31]. Alternatively, the recently developed clone, SIVsm804E-CL757, has been shown to result in SIVE in ~50% of animals around 1 year post infection without immune modulation [39,40]. This clone was developed through serial passages of non-neurovirulent SIVsmE543-3 through RMs [41].

**Table 1:** NHP models of neuroHIV.

Model	Strain	Species	CNS Pathology	SIVE Frequency	Time to SIVE	Reference
**Accelerated CNS disease**	SIV/17E-Fr + SIV/ΔB670	PM	Consistently results in severe SIVE and full immunosuppression	~90%	3–6 months	[26,27,42]
	SIVmac251 + CD8+ T cell depletion	RM	Consistently results in SIVE and full immunosuppression	50–100%	2–6 months	[36,43,44]
**Non-accelerated disease**	SIVmac182	RM	Reliable infection of the CNS, rarely forms encephalitic lesions	Rare	-	[45,46,47]
	SIVmac251	RM	Consistent CNS infection with natural disease progression and reservoir formation	25%	7–36 months	[34,36,48]
	SIVsm804E-CL757	RM	High frequency of SIVE without rapid disease progression	50%	~12 months	[39,40]
	SIVmac239	RM	Consistent CNS infection with natural disease progression and reservoir formation	25%	12–36 months	[34,48,49]
**Simian-HIV chimera**	SHIVSF162P3	RM	Consistent CNS infection with natural disease progression	14%	~6 months	[50]
	SHIV-1157ipd3N4	RM	Reliable infection of the CNS, rarely forms encephalitic lesions	Rare	-	[51]

ART: antiretroviral therapy, CNS: central nervous system, NHP: non-human primate, PM: pigtailed macaque (*Macaca nemestrina*), RM: rhesus macaque (*Macaca mulatta*), SIV: simian immunodeficiency virus, SIVE: SIV encephalitis.

### 2.3. Simian-Human Immunodeficiency Viruses (SHIV)

Whilst the SIV variants above model HIV infection, they still predominantly contain SIV viral genes and proteins, which are evolutionarily different to HIV and differ in their sensitivity to certain antiretroviral (ARV) drugs (i.e., RT-SHIV contains HIVrt genes to ensure sensitivity to nonnucleoside reverse transcriptase inhibitors (NNRTIs), which specifically target HIV-1 reverse transcriptase (RT) and do not effectively inhibit SIV RT [52]). Chimeric/recombinant viruses that infect NHPs and produce HIV proteins, such a HIV-1 env, have been developed to assess vaccine efficacy and broadly neutralizing antibody (bNAb) therapeutic strategies to HIV infection in an NHP setting. The SHIV-1157ipd3N4 infection of the brain has been characterized in RMs and disease progression mimics HIV infection in humans [51], with the viral infection of superficial meninges detected within 12 weeks post infection, with no SIVE lesions present in the CNS [51]. Of note, this strain is R5-tropic and contains a subtype C *env*. SHIVSF162P3 is a CCR5-tropic virus that results in SIVE in 14% of infected animals [50]. Due to the HIV-1 env, both models can be used to assess the effect of novel therapies targeting the HIV envelope.

## 3. SIV Neuropathogenesis

### 3.1. SIV/HIV Entry into the CNS

NHP models of HIV have provided significant insight into early infection events in the brain as studies in PWH are exceptionally difficult and the diagnosis of infection is often made months after transmission events. Furthermore, characterizing viral penetration into the brain can only be defined by CSF viral load testing, which is invasive and may not reflect CNS tissue infection. Human ex vivo studies predict that HIV infects the CNS during early acute infection [53], with loss in the volume of brain parenchyma tissue within the first 100 days of infection [54]. Specifically, HIV-infected CD4+ T cells and monocytes in peripheral blood [55,56,57,58] travel across the blood–brain barrier (BBB) into the CNS through normal immune surveillance [59,60,61], potentially in response to the activation or impairment of the BBB itself. These findings have been supported and extended upon through work in NHPs, where monocytes have been shown to infiltrate/localize within the CNS early during SIV infection [62] and perivascular macrophages are productively infected [63]. Accordingly, the blockade of leukocyte migration across the BBB in SIV-infected RMs via treatment with the anti-α4-blocking antibody natalizumab impeded viral entry into brain and gut tissue compared to untreated SIV-infected macaques [64].

NHP studies have further implicated T cells as a major source of viral infection into the CNS. RMs infected with the non-accelerated SHIV-1157ipd3N4 showed an infiltration of T cells and infected cells in the meninges following 12 weeks post infection [51]. Furthermore, a more recent longitudinal study of SHIV-1157ipd3N4-infected animals detected spliced viral RNA in CD4+ T cells in the CSF, accompanied by a higher frequency of activated CD8+ T cells and monocytes in the CSF, demonstrating the presence of both active viral transcription and likely immune activation during the first 4 weeks of infection [65]. Therefore, HIV/SIV likely enters the CNS via multiple routes and is associated with immune activation during early infection.

### 3.2. CNS Infection, Immune Dysfunction, and Encephalitis during Untreated SIV Infection

Following entry to the brain, HIV/SIV infects long-lived resident CNS cells such as perivascular macrophages, microglia, pericytes, and astrocytes [50,66,67,68,69,70], and SIV DNA persists post acute infection, resulting in the establishment of a brain viral reservoir [71]. Monocyte-derived macrophages and microglia are the main targets for HIV/SIV infection in the CNS. HIV/SIV has also been found to infect astrocytes during acute infection [72,73], which express little to no CD4, supporting the infection of other immune cell types in the brain. Although HIV/SIV does not directly infect neurons, the infection of nearby cells can indirectly cause neuronal death and degradation through the production of inflammatory cytokines (e.g., TNFα) and toxic viral proteins, including tat and refs. [74,75,76,77].

**Figure 1:**
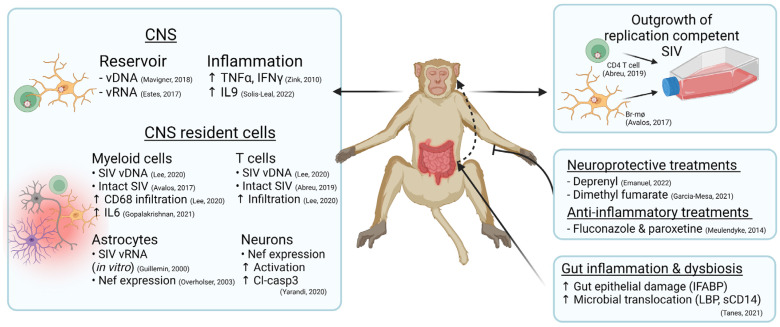
Potential mechanisms driving SIV reservoirs (including replication-competent viruses) and immune dysfunction in the brain of SIV-infected non-human primates.

Acute SIV infection is associated with neuronal damage and activated astrocytes and microglia [78,79]; altered immune processes in the CNS likely also contribute to disease pathology. Specifically, CD8+ T-cell-depleted SIVmac251-infected RMs show the recruitment of MAC387+ macrophages in the meninges and choroid plexus during the first 3–4 weeks of infection [43]. Acute SIV infection is associated with increased IL-6 and pSTAT1 expression, with levels in microglia preceding reliable viral detection within the first week of SIV infection [49,80] (Figure 1). Monocyte/macrophage infiltration is associated with neuronal damage during early SIV infection [44] and the therapeutic blockade of monocyte recruitment into the brain was associated with less neuronal injury [64], supporting a potential proinflammatory effect of myeloid cell accumulation in the brain. These findings in NHP models reflect evidence in ex vivo human studies where well established plasma markers of myeloid cellular activation such as TNFα, IFNγ, sCD14, IL-1β, and IL-6 are generally increased in viremic or ART-naïve PWH and in some cases are associated with HAND severity [81,82,83].

Altered CD8+ T cell responses during acute infection may also contribute to CNS pathogenesis during acute infection. The depletion of CD8+ T cells in multiple SIV NHP models accelerates CNS disease pathology and the administration of anti-CD8 monoclonal antibody in the CSF of SIV-infected animals has been shown to result in an increase in SIV DNA in the brain, and microglial activation [37]. A recent study by Mavian et al. [36] found untreated SIV-infected RMs with detectable brain infection had a significantly higher gene expression of monocyte and macrophage activation markers in animals that had undergone CD8+ T cell depletion. The virus was detected in CSF taken at necropsy; however, the presence of virus in the frontal lobe tissue did not correlate with CSF viral loads. Extensive pathway analysis found 104 differentially expressed genes between animals with and without detectable virus in the brain, including genes integral to the regulatory pathways of reactive oxygen species (ROS) and innate immunity (MIF, C1QB, NCF1, NO) [36].

If left untreated, SIV infection in the CNS results in SIVE, which is characterized by multinucleated giant cells in the brain (i.e., the fusion of multiple cells), mediated by nef [84], neuronal damage, and ultimately death. Encephalitic lesions in the brain form following active viral replication, resulting in the recruitment/possible proliferation of CD163/68+ perivascular macrophages and the recruitment of microglia, CD4+ T cells and B cells [40,43,85]. Furthermore, active viral production and heightened immune activation in SIVE lesions contributes to CNS pathology and neuronal damage. In PWH, HIVE is strongly associated with HIV-associated dementia. Similarly, animals with SIVE have elevated ratios of N-acetylaspartate/creatine, a surrogate measure of neuronal metabolites, during the first month of infection [79]. Specifically, the level of menin in the CNS of SIV- or SHIV-infected animals was increased during infection and was associated with the tat-induced death of neurons, supporting a proapoptotic role of menin in the CNS during SIV infection. Therefore, similar to HIVE, the development of SIVE is associated with death in infected animals.

Finally, similar to findings in PWH, SIV infection is also associated with vascular dysfunction and cerebrovascular disease that may also affect the CNS. SIV infection is associated with endothelial activation and macrophage accumulation around vessels [86]. Furthermore, coagulation markers, including thrombotic microangiopathy in the brain, are predictive of SIV disease [87], indicating a possible role of aberrant coagulation-induced effects on the brains of SIV-infected animals.

### 3.3. SIV/HIV Persistence in the Brain Post-ART

The contribution of the CNS as a long-lived reservoir of replication-competent SIV/HIV viral DNA (vDNA) to ART remains controversial in some circles. This is partly due to the reliance on well characterized autopsy material and difficulties in translating technologies to brain tissues. We and others have shown that brain cells, including microglia/perivascular macrophages, astrocytes, and pericytes from ART-suppressed individuals, harbour HIV DNA [88]. We recently provided the first evidence that ART-suppressed PWH harbour HIV DNA in the brain at similar levels to untreated PWH [89], demonstrating a stable reservoir in the brain. Importantly, we further used droplet digital PCR approaches to identify intact proviral genomes in the brain [89], supporting the presence of potentially replication-competent genomes in the brain. Numerous studies, including our own in human autopsy brain tissue or ex vivo CSF, have detected HIV DNA, which supports the presence of ‘latent’ infection in the CNS compartment [35,90,91]. CNS viral escape has been reported in PWH on virally suppressive ART [92,93,94], where virus was found within the CSF with no detectable virus in peripheral blood, suggesting that the CNS viral reservoir may re-seed the periphery as virus can exit the brain [92]. However, ex vivo studies are limited by accessibility issues and confounding factors. Furthermore, the impact of the CNS viral reservoir on viral rebound after treatment cessation is controversial (as discussed in [95]).

NHP studies have further advanced our understanding of a viral reservoir in the brain of ART-suppressed animals (Table 2). A comprehensive full-body analysis of SIV/SHIV-infected macaques virally suppressed with ART found cell-associated viral RNA (vRNA) in every organ system tested, including the brain, despite undetectable virus in peripheral blood [35]. This study included SIVmac251 and RT-SHIV-infected RMs which were either left untreated, or treated with suppressive ART. The tissue viral load of the lymph node, gut, spleen, brain, kidney, heart, lung, and liver was quantified using RNAscope/DNAscope and the found frequency of infected cells in virally suppressed animals was lowered in lymph node tissue, and to a lesser extent in gut and spleen tissue. However, the frequency of vRNA+ cells in the brain tissue was not reduced, likely due to the poor penetration of ART into the brain [35]. These findings are supported by additional work using classic in situ hybridization (ISH) or RNAscope/DNAscope to detect single copies of vRNA or vDNA, which have consistently detected SIV in multiple brain regions, including the basal ganglia, frontal, and parietal cortex, despite ART treatment [30,35,96,97,98,99,100] (Figure 1).

Persistent vDNA+ cells are found in tissue reservoirs in the CNS and periphery, likely due to the inability of ART to target latent virus. The SIV brain reservoir was also demonstrated in the SIVE NHP model of the dual inoculation of SIV/ΔB670 and SIV/17E-Fr in pigtailed macaques [101]. All animals in this study were given ART, with seven of the eight animals reaching viral suppression. All animals had vDNA+ cells detected in the CNS; however, vRNA was not detected in the virally suppressed animals, indicating a latent viral reservoir [101]. Mavigner and colleagues found no changes in vRNA or vDNA in the CNS of ART-suppressed NHPs compared to viremic controls [98]. Furthermore, ART (tenofovir, emtricitabine, and dolutegravir) concentrations were not detected in all brain sections measured [98]. An early study of the effects of ART in the CNS found no reduction of vDNA between treated and untreated NHPs; however, vRNA levels were undetectable [29].

**Table 2:** Evidence of a SIV/SHIV reservoir in the brain of NHPs.

Study	*n*	Virus	Inoculation Route	WPI	ART (wks)	CNS Infection (vDNA or vRNA+)	Technique	Tissue
Estes, et al. [35]	5	SIVmac251/RT-SHIV	i.v.	28–30	20–26	Yes (vRNA)	RNAscope/DNAscope	Cerebrum
Hsu, et al. [51]	12	SHIV-1157ipd3N4	IR or IV	12	No ART	Yes (vRNA)	RNAscope	Meninges
Hsu, et al. [102]	4	SHIV-1157ipd3N4	IR	18	16	No	RNAscope	Posterior cingulate gyrus
Yarandi, et al. [97]	3	SIVmac251 (with CD8 depletion)	i.v.	17	14	Yes (vRNA)	RNAscope	Hippocampus
Mavigner, et al. [98]	4	SIVmac251 (infant)	Oral	31–42	26–37	Yes (vRNA and vDNA)	RNAscope/DNAscope	FC, PC and BG
	12	SIVmac251	i.v.	25–69	24–61	Yes (vRNA and vDNA)	RNAscope/DNAscope	FC, PC and BG
Bissel, et al. [99]	5	SIVmac251	i.v.	48–63	10–25	Yes (vRNA)	ISH	MFC, caudate, putamen, hippocampus and cerebellum
Abreu, et al. [103]	4	SIVmac251	i.v.	19	17	Yes (vRNA and vDNA)	qPCR, ddPCR, QVOA	Macrophage isolated from FC, PC, TC, BG and TH
Zink, et al. [29]	5	SIV/17E-Fr + SIV/ΔB670	i.v.	23–25	21–23	No (vRNA)Yes (vDNA)	Real time PCR and RT-PCR	BG
Gama, et al. [30]	5	SIV/17E-Fr + SIV/ΔB670	i.v.	33–90	28–71	Yes (vRNA)	RNAscope and ddPCR	OC, BG and PC
Avalos, et al. [101]	8	SIV/17E-Fr + SIV/ΔB670	i.v.	28–91	26–89	No (vRNA)Yes (vDNA)	ISH and qVOA	OC, BG and PC
Lee, et al. [40]	5	SIVsm804E-CL757	ND	8–15	No ART	Yes (vRNA and vDNA)	qPCR, coculture, RNAscope	Isolated mononuclear cells and midbrain

ART: antiretroviral therapy, BG: basal ganglia, ddPCR: digital droplet PCR, FC: frontal cortex, IR: intrarectally, i.v: intravenous, IV: intravaginally, ISH: in situ hybridization, MFC: midfrontal cortex, ND: not described, OC: occipital cortex, PC: parietal cortex, qPCR: quantitative PCR, qVOA: quantitative viral outgrowth assay, RT-PCR: reverse transcription PCR, SIV: simian immunodeficiency syndrome, TC: temporal cortex, TH: thalamus, vDNA: viral DNA, vRNA: viral RNA, WPI: weeks post infection.

Whether viruses present in the CNS are truly replication-competent and capable of producing infectious virions, thus contributing to subsequent cellular infection, is a major question in the field and exceptionally difficult to answer in humans ex vivo. Studies in human peripheral blood mononuclear cells (PBMCs) have demonstrated that the large majority of proviruses are replication incompetent (i.e., ‘defective’) due to hypermutations and/or deletions in the genome [104]. Therefore, quantifying the total reservoir size is misleading as it likely overestimates the total number of intact, replication-competent genomes.

Alternatively, assays that measure functional HIV transcripts, full length genomes, viral proteins, or ultimately viral outgrowth in target cells are more indicative of the replication competence of viral reservoirs. Ex vivo human studies have detected HIV RNA in the CSF of ART-suppressed individuals, potentially related to ongoing viral replication in the brain. However, it is possible that these viruses are entering the CSF from the blood, and are therefore not truly reactivating viruses from the CNS. To date, the best evidence of possible replication-competent viral genomes in the CNS derives from NHP studies. A study using pigtailed macaques infected with SIV/ΔB670 and SIV/17E-Fr were virally suppressed for 500 days with ART followed by latency reversal treatment demonstrated increased CSF viral loads, supporting the activation of latent virus present in the CNS [30]. In another study, a macrophage quantitative viral outgrowth assay was developed to demonstrate that the majority of virally-suppressed animals contained latently infected brain macrophages, and that virus produced in the outgrowth assay was replication competent [101]. Therefore, the CNS viral reservoir represents a barrier for both the long-term treatment of HIV and cure research, due to the potential reseeding of CNS virus into the periphery after ART cessation, as well as the local immune environment in the CNS during ART suppression.

### 3.4. Chronic Immune Activation Is Present in the CNS of ART-Suppressed PWH and SIV-Infected NHPs

Chronic immune activation and cellular dysfunction in the CNS likely persists following ART and is hypothesized to contribute to the pathogenesis of HANDs in ART-suppressed PWH. The low-level expression of the HIV tat protein promotes a neurodegenerative phenotype caused by a reduction in brain volume, astrocyte activation, inflammatory cytokine expression, as well as synapse and axonal damage [77,105,106]. Immune activation markers including sCD163, sCD14, neurofilament light chain (NF-L), glutamate, neopterin, CXCL-10, high mobility group box 1 (HMGB1) and IL-8 remain elevated in the plasma/CSF of ART-suppressed PWH [107,108,109,110,111,112,113], suggestive of ongoing neuroinflammation and neuronal damage in these individuals. Furthermore, plasma sCD163 levels distinguish the milder forms of HANDs in virally suppressed individuals [114]. However, these findings are limited in that they assess the surrogate biomarkers of neuroinflammation measured in the CSF/plasma, which may not necessarily reflect ongoing immune activation in the brain and may be confounded by immune activation systemically.

To date, comparatively few studies have assessed the immune environment in the brain of ART-suppressed SIV-infected animals, and findings appear to differ depending on the models used. As described above, untreated SIV infection generally is associated with elevated immune activation, oxidative stress, and cellular dysfunction. Several studies using accelerated infection models show that ART treatment reduces the level of inflammatory cytokines compared to untreated SIV infection; however, levels remain elevated in ART-treated animals relative to SIV-uninfected controls [29,115]. Specifically, the levels of macrophage infiltration (CD68) and TNFα and IFNγ staining by immunohistochemistry were not significantly changed within the CNS, indicating ongoing cellular activation in the brain [29]. Increased measures of cell death (as measured by cleaved-caspase 3 staining) in neurons has also been detected in the CNS of ART-treated NHPs [97]. Importantly, cleaved-caspase 3 was co-expressed in Nef+ neurons in ART-treated animals [97] (Figure 1), highlighting the cascading effect of viral proteins on the neuroimmune environment leading to neuronal death [75]. Solis-Leal and colleagues also identified the higher gene expression of proinflammatory IL-16, IL-6R, and IL-9, and chemokines CX3CL1 and CXCL12, in the basal ganglia tissue of SIVmac251-infected Chinese-origin RMs on suppressive ART [115] (Figure 1).

Conversely, several studies have found that ART treatment results in the near complete resolution of heightened immune activation in the brain. Mx2, superoxide dismutase 2 (SOD2), TNFα, and CCL2 levels in the brain of ART-treated SIV-infected RMs were similar to uninfected controls in contrast to heightened levels in ART-naïve animals, supporting the idea that ART treatment dampens SIV-infected immune activation in the brain [100,116,117]. These findings have been further supported by a recent study using single-cell RNAseq that identified that the inflammatory profile of microglia isolated from ART-suppressed SIVmac251-infected animals were more similar to those seen in uninfected animals than untreated SIV-infected animals [118]. Therefore, ART-treatment likely dampens immune activation in the brain.

## 4. Confounding Drivers of SIV Neuropathology

### 4.1. Chronic Systemic Inflammation

The gut is one of the first major sites of viral replication following HIV infection due to its high density of HIV susceptible CD4+ T cells [119,120]. The preferential destruction of approximately 60% of CD4+ T cells and the ensuing inflammatory response in the gut causes damage to the epithelium [121,122], leading to microbial translocation and the perpetuation and augmentation of immune activation and inflammatory sequela that further damages the gut. Seminal studies by Estes, Brenchley, and colleagues identified the effects of SIV-induced gut damage on SIV disease pathogenesis, which had significant implications on PWH [123]. SIV infection in RMs mimics the key pathological features of the natural progression of HIV in humans, such as CD4+ T cell depletion, chronic systemic inflammation, lymphoid and GIT tissue pathology, neuropathology, establishment of the latent reservoir, and progression to AIDS (as reviewed in [22]). Sooty mangabeys and African green monkeys are natural hosts of SIV and therefore do not progress to AIDS, despite high viremia and active viral replication [121,122]. Both progressive and non-progressive NHP models of SIV have contributed to the discovery and establishment of key mechanisms of HIV pathogenesis, including the critical role GIT damage and microbial translocation have in driving systemic inflammation [119,123].

This physical and immunological damage to the gut impairs mucosal integrity resulting in a “leaky gut” whereby microbes and their products such as lipopolysaccharide (LPS) can translocate through the enterocytes and between epithelial cells into the blood stream. The resulting gut dysbiosis persists despite ART [124,125,126], likely due to the inability to restore the normal colonic immunologic environment within the gut [127]. Microbial translocation, as measured by plasma levels of LPS and soluble LPS coreceptor levels (sCD14), has been found to be an early predictor of HAND progression [83,128,129,130,131] and HIV-related mortality [132] in ART-treated PWH. Numerous studies have consistently documented GIT epithelial barrier damage (e.g., levels of intestinal fatty-acid binding protein (I-FABP)), inflammation, and dysbiosis that occurs during SIV infection [123] (as reviewed in [133]). However, data of persistent inflammation and microbial translocation in ART-treated animals have generated varied results. Longitudinal microbiome studies of ART-treated RMs demonstrate the potential reversal of microbial dysbiosis after long-term viral suppression [133,134,135]. While partial recovery is seen in the gut microbiome, biomarkers of epithelial barrier damage and microbial translocation remain elevated [136,137] (Figure 1). Therefore, further research is required to establish how bacterial taxa can impact SIV pathogenesis and systemic inflammation.

### 4.2. Limited Penetrance and Toxicity of ART

Studies in humans indicate that some ART compounds do not penetrate the brain, which may lead to pockets within deep tissue sites including the brain where HIV/SIV may be able to replicate, drive immune activation, and contribute to neurocognitive decline [77,93,138]. In addition to the CNS, lower antiretroviral drug levels were found in lymphatic compartments compared to peripheral blood, which may contribute to the formation and persistence of viral reservoirs within these peripheral compartments [139,140]. However, to date, limited evidence of ART penetration in NHP models exists. Similar to human studies, we demonstrated that ART penetrance in lymph node, ileum, GALT, and RALT in SIV-infected animals was lower compared to blood [35]. However, the penetrance of ART into the brain was not assessed in this study.

### 4.3. Substance Use, Ageing and Other Modifiable Risk Factors

PWH have higher rates of illicit drug use than HIV-seronegative individuals, which may potentiate HAND pathogenesis. Importantly, the use of SIV animal models has allowed for the controlled assessment of each modifiable risk factor on neuropathogenesis, where morphine use, methamphetamine use, smoking, and even age have been linked to neuropathogenesis in SIV-infected animal models. Specifically, morphine use accentuates SIV neuropathology including higher levels of the infiltrating of myeloid cells in the brain during acute infection relative to SIV-infected animals who were not treated with morphine [141]. Interestingly, methamphetamine and/or morphine use are also associated with higher levels of SIV vRNA and immune activation in the brain of SIV-infected animals relative to SIV-infected animals not treated with either drug [142,143,144,145]. Conversely, cocaine use does not appear to impart significant effects of either SIV reservoir size or immune activation in the brain [146].

## 5. Can We Model SIV/HIV-Related Cognitive Impairment in NHPs?

Cognitive and behavioral testing is essential in characterizing the pathogenesis of the milder forms of HANDs. However, the evidence of the suitability and applicability of NHP models of HIV to study cognitive impairment is limited. NHPs infected with neurovirulent strains of SIV (SIV/17E-Fr + SIV/ΔB670) and experimentally induced encephalitis have shown deficits in motor control and spatial working memory with similarities to PWH [147,148,149,150]. Deficits in motor (forearm force) and cognitive (progressive ratio tests) function associated with SIV infection have been associated with CSF viral load and have also been shown to be exacerbated by morphine treatment [151]. However, as virologically well controlled PWH rarely experience severe dementia or HIVE, the relevance of these outcomes in NHPs or the suitability of these cognitive tests as a measure of more mild impairment are unclear. A study of Chinese-origin RMs infected with SIVmac251 and either suppressed with ART or left untreated found no differences in cognitive function, despite detectable viral load in the CSF [99]. However, it is of note that these animals were aged and showed no changes in inflammatory cytokine levels or SIV-induced disease pathogenesis, possibly supporting a role for immune activation in contributing to HAND pathogenesis. Alternatively, RMs infected with neurovirulent SIVmacR71/E17 and treated with morphine showed impaired behavioral deficit relative to SIV-infected animals [151].

Broader behavioral testing used in NHP models of other cognitive disorders in the absence of encephalitis may offer translational benefit for modelling mild HANDs. In humans, HAND diagnosis includes a battery of cognitive testing that spans multiple cognitive domains including: verbal/language; attention/working memory; abstraction/executive; memory (learning, recall); speed of information processing; sensory–perceptual; and motor skills [6]. Although the specific test may change, each cognitive domain can be measured and compared between humans and NHPs. The 5-Choice Reaction time task is a well-defined model of attention that has been tailored for use in humans, NHPs, and rodents (as reviewed in [152]). This model primarily measures sustained attention through a brief visual stimulus and the reaction times over consecutive trials (generally 80–100 trials per session).

In contrast to SIV models, behavioral testing in NHP models of other neurocognitive disorders such as Huntington’s and Parkinson’s disease are more well-established and may be applicable to chronic SIV. Specifically, cognitive impairment and anxiety-like behavior have been described in a transgenic NHP model of Parkinson’s disease [153]. Behavioral testing protocols relating to age-related learning and function may also be translated to SIV infection as age-associated decline in cognitive and motor function, including delay in learning in simple or precision-based tasks relative to younger animals has been measured in naturally ageing baboons (~20 years old) [154]. Thus, it is possible that the translation of the measures of behavioral changes in other neuroinflammatory disorders such as MS or even healthy ageing may have some utility in assessing SIV-induced cognitive impairment.

## 6. Targeting SIV Reservoirs and Chronic Inflammation in the Brain

One major benefit of utilizing NHP models of SIV/SHIV infection is the flexibility to perform intervention studies to target SIV brain infection and/or inflammation. Reactivating latent HIV in circulating CD4+ T cells as part of ‘shock and kill’ strategies have been tested in vitro and in vivo in NHPs. However, comparatively little is known regarding targeting reservoirs in the brains of SIV-infected animals.

The treatment of SIV-infected animals with the second mitochondria-derived activator of caspases (SMAC) mimetic AZD5582 induced reactivation of viral reservoirs in the blood and tissue of ART-treated animals [155]. Whilst brain tissue was not assessed from these animals, related experiments in rodent models supported viral reactivation in the CNS, potentially supporting the efficacy of these treatments in NHPs. As a complementary therapy, HSC-derived, virus-targeting CAR-T cells that traffic to, and persist within, the brain may offer an opportunity to target reactivating cells; as a recent study by Barber-Axthelm shows, this therapeutic modality shows significant promise in targeting brain- and CNS-resident reservoirs, in addition to reservoirs located within classic peripheral lymphoid sites [156].

Another cutting-edge technique that highlights the necessity of using NHP models for developing novel R&D is a recent proof-of-concept for utilizing CRISPR/Cas9 to reduce the amount of proviral DNA present within the brain and CNS (among other sites; [157]). With efficiencies reaching up to 100% in select sites within the CNS and brain, including the frontal lobe, CRISPR/Cas9 shows promise in reducing the symptoms/progression of HANDs stemming from local inflammation as a result of the limited reactivation of replication-competent SIV/HIV, especially when considered in combination with treatments targeting other sources of neuroinflammation (below).

Treatments selectively targeting neuroinflammation and oxidative stress in the brain have had some benefit in SIV-infected NHPs. Meulendyke and colleagues identified fluconazole and paroxetine (FluPar) as protective compounds against gp120 and tat neurotoxicity (Figure 1). This treatment commenced during early acute infection to pigtail macaques infected with accelerated neurovirulent SIV. Interestingly, FluPar treatment provided neuroprotection, as measured by CSF neurofilament light chain and frontal cortex CaMKIIα, despite unchanged levels of neuroinflammation [158]. Treatment with the monoamine oxidase inhibitor, deprenyl, also showed some benefit in reducing peripheral and CNS inflammation when administered during acute SIV infection [159]. Specifically, deprenyl treatment decreased both peripheral and CNS inflammation isolated from frontal grey matter, caudate, hippocampus, and spleen tissue. Although no changes in viral load were detected with deprenyl treatment, the downregulation of inflammatory genes was only observed in SIV-infected animals, indicating a specific response to SIV-associated neuroinflammation [159]. Other repurposed drugs, such as Dimethyl fumerate, used in MS treatment, were found to decrease brain oxidative injury and inflammation in SIV-infected RMs [160].

Finally, targeting systemic inflammation, characteristic of chronic HIV/SIV infection, may also be of benefit in improving neuroSIV pathogenesis. The treatment of SIV-infected animals with drugs designed to target microbial translocation via controlling microbes or gut inflammation (rifaximin and sulfasalazine, respectively) showed benefit in lowering systemic immune activation and microbial translocation during acute infection [161]; however, the effects on chronic inflammation or neuroinflammation are unclear. Further understanding the role of chronic systemic inflammation on neuroinflammation and cognitive dysfunction are required.

## 7. Conclusions

The mechanisms contributing to viral persistence, neuroinflammation, and cognitive disorders in PWH remain a tangled web that is difficult to delineate in human ex vivo studies alone. Thus, clinically and physiologically relevant NHP models are essential tools required to define the relationship between mild neurocognitive disorders, CNS viral burden, systemic inflammation, and immune activation in the brain. Understanding the roles and contributions of each element to ongoing brain disease will guide further therapeutic interventions with translational capacity for people living with chronic HIV and HANDs.
